# Intraoperative Hyperglycemia Augments Ischemia Reperfusion Injury in Renal Transplantation: A Prospective Study

**DOI:** 10.1155/2011/652458

**Published:** 2011-09-04

**Authors:** Justin Parekh, Claus U. Niemann, Kim Dang, Ryutaro Hirose

**Affiliations:** ^1^Department of Surgery, Division of Transplantation, University of California San Francisco, San Francisco, CA 94143, USA; ^2^Department of Anesthesia and Perioperative Care, University of California San Francisco, San Francisco, CA 94143, USA; ^3^Stanford University School of Medicine, Stanford, CA 94305, USA

## Abstract

*Background*. Diabetes is a risk factor for delayed graft function in kidney transplantation, and hyperglycemia increases ischemia reperfusion injury in animal models. *Methods*. To explore the role of perioperative hyperglycemia in ischemia reperfusion injury, we conducted a prospective study of 40 patients undergoing living donor renal transplantation. Blood glucose levels were monitored intraoperatively, and serum samples were obtained at the time anesthesia was induced and one hour after allograft reperfusion. The percentage change in neutrophil gelatinase-associated lipocalin (NGAL), a protein whose expression is increased with renal ischemia, was then used to determine the extent of injury. *Results*. In a multivariate model including recipient, donor, and transplant factors, recipient blood glucose >160 mg/dL at the time of allograft reperfusion (*β* 0.19, *P*-value < 0.01), warm ischemia time >30 minutes (*β* 0.11, *P*-value 0.13), and recipient age (*β* 0.05, *P*-value 0.05) were associated with percentage change in NGAL. These same predictors were associated with the percentage change in creatinine on postoperative day 2. *Conclusions*. Hyperglycemia is associated with increased ischemic injury in renal transplantation. Both creatinine and NGAL, a marker of ischemic injury and renal function, fall less rapidly in patients with elevated blood glucose.

## 1. Introduction

The success of renal transplantation continues to be limited by ischemia reperfusion injury, which is the dominant mechanism in the development of delayed graft function (DGF). Commonly defined as the need for dialysis within seven days of transplant, DGF is greatly influenced by factors that lead to greater ischemic injury, such as prolonged cold storage and procurement from expanded criteria or nonheart beating donors. Beyond the need for short-term dialysis, DGF affects long-term outcomes and has been associated with a greater incidence of rejection and decreased graft survival [1–4]. 

Recently, diabetes has emerged as another risk factor for DGF [5, 6]. Higher glucose levels in donors have also been linked to worse prerecovery renal function [7]. In animal models, hyperglycemia has been shown to increase ischemia reperfusion injury. For example, hyperglycemic diabetic mice suffer larger myocardial infarcts, and rabbits with tight glycemic control have less spinal cord injury than hyperglycemic mice [8–10]. In addition, we have shown that hyperglycemia at the time of warm ischemia increases renal injury in nondiabetic Lewis rats [11]. In our experiments, rats with hyperglycemia at the time ischemia was induced had higher terminal creatinine levels and histological features of more severe acute tubular necrosis compared to normoglycemic rats. However, these differences were not observed when hyperglycemia was induced two hours after ischemia was induced. These findings indicate that hyperglycemia increases ischemia reperfusion injury, and that this effect may be limited to a window around the time of injury. 

Although the extent of ischemic injury has been documented in animal models, demonstrating similar results in humans can be challenging because of the risk of obtaining tissue for direct pathologic examination. However, novel biomarkers have been developed that correlate well with acute kidney injury due to ischemic insults [12]. Neutrophil gelatinase-associated lipocalin (NGAL) is emerging as an excellent marker of acute kidney injury. NGAL is 25-kDa protein that is covalently bound to gelatinase from neutrophils. Normally found at low levels in multiple tissues, its expression is increased in direct response to ischemic injury. In particular, NGAL is one of the earliest and most strongly expressed proteins in the kidney after ischemic injury [13–15]. Increased NGAL expression has been detected as early as two hours after cardiopulmonary bypass in patients who go on to develop acute kidney injury [16, 17]. NGAL levels have also been reliably linked to DGF and the degree of allograft injury in renal transplantation. NGAL expression in biopsy specimens from renal allografts taken one hour after re-perfusion is strongly correlated with peak serum creatinine and DGF [18]. Similarly, urine NGAL levels on the day of surgery clearly identified kidney transplant recipients who went on to develop DGF, with an overall area under the curve of 0.9 [19]. More recently, NGAL levels have been found to start at elevated levels in kidney transplant recipients and stayed elevated in patients who would develop DGF, thereby, supporting the use of the change or relative change in NGAL as a marker for injury [20].

Given the role of recipient diabetes in DGF [5, 6] and mounting evidence that hyperglycemia increases ischemia reperfusion injury, we undertook this study to determine if intraoperative hyperglycemia increases ischemic injury in renal transplantation using serum NGAL as our primary outcome.

## 2. Subjects and Methods

### 2.1. Study Design and Patients

We prospectively enrolled a cohort of 40 adult patients undergoing living donor renal transplantation and their donors at the University of California, San Francisco (UCSF) between December 1, 2008 and June 15, 2009. The study was approved by the Committee on Human Research at UCSF, and informed consent was obtained. One patient was dropped from the study because of enrollment in a concurrent experimental drug trial. We selected living donor transplantation because it is performed on an elective basis, thereby, allowing us to minimize the effect of cold ischemia time and donor and intraoperative factors. All organ procurement was completed via laparoscopic donor nephrectomy [21]. The allograft was then put on ice and transported to the recipient operation where it was flushed and prepared for transplantation. After giving each recipient 500 mg of methylprednisolone and either rabbit antithymocyte antibody (Thymoglobulin, Genzyme Corporation, Cambridge, Mass, USA) or basiliximab (Simulect, Novartis, East Hanover, NJ) as induction immunosuppression, the graft was anastamosed to the external iliac arteries and veins via an extraperitoneal approach. Intravenous furosemide and mannitol were infused before graft reperfusion. Warm ischemia time was equivalent to anastomotic time. 

Although NGAL levels are an excellent marker of acute injury and predictor of DGF, they can change quickly and are variable between individual recipients. Therefore, we used the percentage change in serum NGAL level as our primary outcome. In particular, we measured a baseline level just after the induction of anesthesia to minimize external factors and focused on the percentage change in NGAL in order to negate the variability between patients.

### 2.2. Data Collection

Blood samples were obtained from the recipients after anesthesia was induced (baseline) and one hour after the reperfusion of the allograft. Serum samples were then aliquoted and stored at −20°C. Blood glucose concentrations were determined at the point of care at baseline, the time of reperfusion, and one hour after reperfusion via glucometer. Data was collected on recipients (age, gender, body mass index (BMI), diabetes, cause of renal failure, panel reactive antibody (PRA), number of previous transplants), donors (age, gender, BMI), and transplant factors (warm ischemia time, induction immunosuppression regimen, and volume of intraoperative intravenous fluids(IVF)).

After transplantation, serum creatinine was measured on each postoperative day (POD) while the patient was hospitalized. Patients underwent follow-up for 90 days and data on any episodes of rejection and a follow-up creatinine at 90 days (±7 days) were recorded.

NGAL levels were determined via enzyme-linked immune-absorbance assay (ELISA) kits commercially prepared by R and D systems (Minneapolis, Minn, USA). The specimens were then grouped into batches of 10 and tested in duplicate every month such that no specimen was stored for more than one month. Controls were sampled from healthy volunteers with no renal disease (*n* = 5). Color intensity was read at 450 nm with wavelength correction at 570 nm on the Tecan Infinite 200 Plate Reader (Tecan, Männedorf, Switzerland). The *R* ^2^ for the standard curve on every plate exceeded 0.99.

### 2.3. Statistical Analysis

All categorical values were reported as simple percentages, and all continuous variables were reported as means ± standard deviations. 

To determine the factors that affect the percentage change in NGAL at one hour, the aforementioned donor, recipient, and transplant variables were analyzed using univariate linear regression. Recipient blood glucose was analyzed as both a continuous and dichotomous predictor (defined as blood glucose >160 at the time of reperfusion). Given our clinical suspicion and evidence of greater injury with blood glucoses exceeding 160 mg/dL, our final models incorporated hyperglycemia as a dichotomous predictor. Because we were interested in ischemic injury, both intra-operative glucose level and warm ischemic time were included in the final multivariate model. Interaction terms were not explored based on a lack of clinical suspicion. The final multivariate model was then created by backwards stepwise elimination with a *P*-value of 0.2 as the cut-off. This technique for predictor selection was also applied to model the percentage change in serum creatinine on POD 2. Model diagnostics were checked to ensure that the underlying assumptions of linearity, normality, and constant variance were not violated. 

Correlations between the percentage change in NGAL and changes in serum creatinine were tested with Spearman's correlation coefficients. STATA 10 statistical software package was used for all calculations. All *P* values were two tailed and *P* values ≤ 0.05 were considered statistically significant.

## 3. Results

### 3.1. Donor and Recipient Characteristics

Data was obtained for 39 living donors who were all healthy volunteers without any comorbid conditions. Forty-six percent were men, the average age was 40.79 ± 11.17 years, and the average BMI was 26.03 ± 3.56 kg/m^2^. Most donors were Caucasian (54%), followed by Hispanic (25%), Black (13%), and Asian/Pacific Islander (8%). 

Data was obtained for 39 recipients, 51% of whom were men. The average age was 47.41 ± 12.78 years, and the average BMI was 26.30 ± 5.09 kg/m^2^. Recipients were most commonly Caucasian (54%) followed by Hispanic (28%), Black (13%), and Asian/Pacific Islander (2%). Forty-six percent were diabetic. The recipients also had an average panel reactive antibody of 11% and 15% of them had a prior kidney transplant. Twenty-six percent of recipients had a warm ischemic time in excess of 30 minutes and received an average of 3.30 ± 0.85 liters of IVF intraoperatively. Induction immunosuppression consisted of either Thymoglobulin (33%) or Simulect (66%). 

Post-operatively, there were no recorded episodes of hypotension (systolic blood pressure <100 or mean arterial pressure <60 mmHg). None of the recipients required dialysis prior to discharge.

### 3.2. Glucose Levels during Transplant

**Figure 1:**
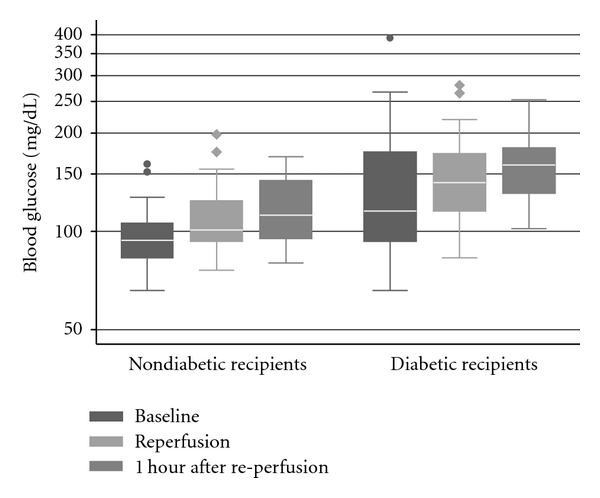
Pattern of intra-operative glucose levels for diabetic (*n* = 18) and nondiabetic patients (*n* = 21) during kidney transplantation. Whiskers span the 5th and 95th percentiles of all data. Dots represent outliers.

Intra-operative glucose levels consistently increased from the beginning to end of the case for both diabetic and nondiabetic recipients (Figure 1). Two patients received intra-operative insulin infusions, and three additional patients received bolus insulin during their operations. Even when patients who were treated for hyperglycemia were included, diabetic recipients had higher glucose levels initially (99.29 versus 144.83 mg/dL, *P* = 0.02), at the time of reperfusion (114.29 versus 155.61 mg/dL,  *P* < 0.01), and one hour after reperfusion (119.52 versus 160.83, *P* < 0.01). However, nondiabetics were still prone to hyperglycemia with 7 (33%) recipients reaching a serum blood glucose of >120 mg/dL at the time of reperfusion, with a peak value of 198 mg/dL in one non-diabetic patient.

### 3.3. Predictors of Percentage Change in NGAL

**Figure 2:**
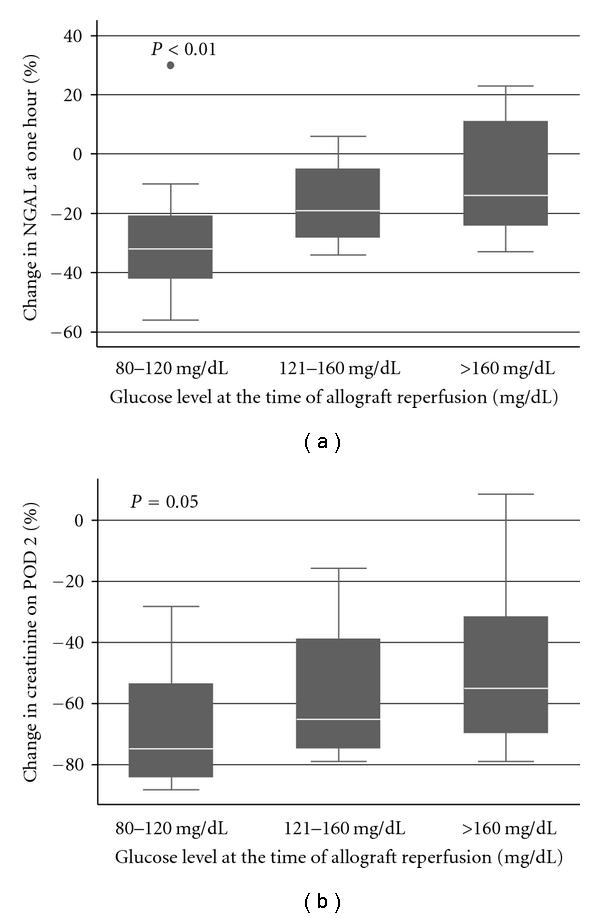
(a) Percentage change in NGAL levels at one hour by clinically defined glucose level (80–120 mg/dL, *n* = 15; 121–160 mg/dL, *n* = 14; and >160 mg/dL, *n* = 10) at the time of allograft reperfusion. (b) Percentage change in creatinine on POD 2 by clinically defined glucose level (80–120 mg/dL, *n* = 15; 121–160 mg/dL, *n* = 14; and >160 mg/dL, *n* = 10) at the time of allograft reperfusion. Statistical significance determined by analysis of variance. Whiskers span the 5th and 95th percentiles of all data. Dots represent outliers.

Compared to controls (42.35 ± 17.31 ng/mL, *n* = 5), recipient NGAL levels were elevated at baseline (639.77 ± 270.66 ng/mL) and fell, on average, one hour after reperfusion (488.43 ± 210.82 ng/mL). However, NGAL levels fell less rapidly and even increased as recipient blood glucose at reperfusion increased (Figure 2(a)).

Univariate linear regression revealed that recipient age, blood glucose at the time of allograft reperfusion (both continuous and dichotomous predictors) and recipient diabetes were all statistically significant predictors of the percentage change in NGAL (Table 1(a)). Warm ischemia time, recipient gender, recipient BMI recipient race, total IVF, induction immunosuppression, donor age, donor gender, donor race, and donor BMI were not significant predictors.

When each of the variables from the univariate analysis was incorporated into a multivariable model and then eliminated in a stepwise fashion, recipient blood glucose >160 mg/dL emerged as a statistically significant predictor of the percentage change in NGAL (Table 1(b)).

### 3.4. Creatinine as a Marker of Renal Function

Recipient creatinine decreased on average from POD 0 (6.81 ± 2.78 mg/dL) to POD 2 (2.57 ± 1.64 mg/dL). Once again, serum creatinine fell less rapidly as blood glucose increased (Figure 2(b)).

Similar to our NGAL analysis, we used both univariate and multivariate linear regression to analyze how blood glucose at reperfusion affected the percentage change in creatinine, a more traditional marker of renal function. Univariate analysis revealed that blood glucose at reperfusion, recipient age, recipient diabetes, recipient BMI, donor age, induction immunosuppression, and warm ischemia time >30 minutes were all statistically significant predictors of the change in creatinine on POD 2. Recipient gender, recipient race, PRA, total IVF, donor gender, donor race, and donor BMI were not statistically significant predictors of the percentage change in creatinine (Table 2(a)).

The final multivariate model was then created. Once again, blood glucose >160 mg/dL at reperfusion, warm ischemia time >30 minutes, and recipient age were all statistically significant. Recipient BMI also emerged as a predictor of the change in creatinine in our final model (Table 2(b)).

Similarly, we calculated correlation coefficients to see how well the percentage change in NGAL correlated with changes in recipient creatinine. We found that percentage change in NGAL at one hour showed good correlation with the percentage change in creatinine on POD 2 (Rho = 0.38, *P* = 0.02).

### 3.5. Renal Function at 90 Day Follow-Up Visit

**Figure 3:**
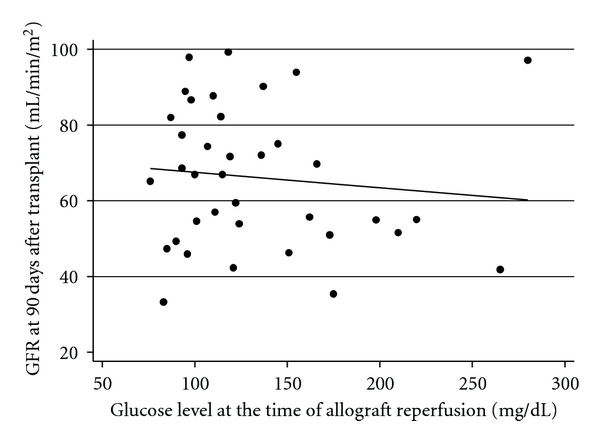
Scatter plot of blood glucose at the time of reperfusion against GFR at 90 days after transplant. Dots represent data points. Solid line represents linear line of best fit.

At the 90-day follow-up visit after transplant, every recipient had a functioning allograft with a mean glomerular filtration rate (GFR) of 66.14 ± 18.65 mL/min/1.73 m^2^. Of the 38 patients with follow-up data, 17 (45%) had a GFR of less than 60 mL/min/1.73 m^2^. Using logistic regression, we found that a glucose exceeding 160 mg/dL at the time of allograft reperfusion was associated with a GFR of less than 60 mL/min/1.73 m^2^ (odds ratio 7.78, 95% CI 1.34 to 45.09, *P*-value 0.02). Due to a limited number of recipients with GFR less than 60 mL/min/1.73 m^2^, we were not able to perform a more complex multivariable analysis. When analyzed as continuous variables, higher blood glucose at the time of reperfusion was associated with lower GFR at 90 days. However, this relationship was not statistically significant (*β* = −0.41, 95% CI − 1.68 to 0.87, *P*-value 0.51) (Figure 3). 

**Figure 4:**
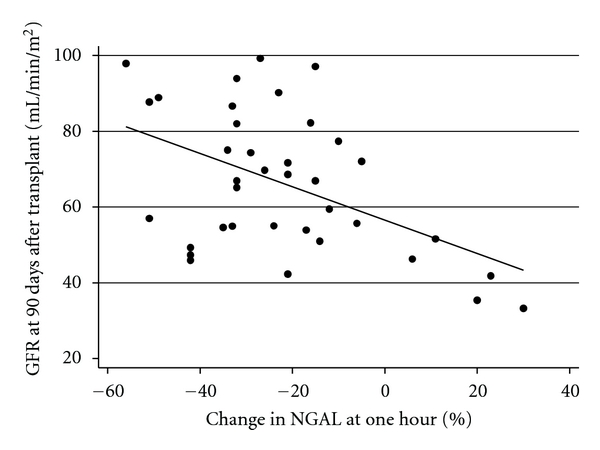
Scatter Plot of percentage change in NGAL at one hour against GFR at 90 days after transplant. Dots represent data points. Solid line represents linear line of best fit.

Similarly, we set out to determine if recipient GFR at 90 days was related to the percentage change in NGAL at one hour. Linear regression demonstrated that a more rapid decline in NGAL at one hour was strongly correlated with the GFR at 90 days (*β* = −0.53, 95% CI −0.86 to −0.020, *P* < 0.01) (Figure 4).

## 4. Discussion

The results of our study elucidate the role of hyperglycemia in ischemia reperfusion injury during renal transplantation and have clinical implications for the perioperative management of glucose levels. Furthermore, our findings provide more information for the use of serum NGAL as a diagnostic tool. 

This study is the first to document intra-operative trends in blood glucose levels during renal transplantation for both diabetic and non-diabetic recipients. While diabetic recipients were clearly at greater risk for intra-operative hyperglycemia, 33% of non-diabetic recipients reached a blood glucose exceeding 120 mg/dL. These patterns imply more rigorous glucose monitoring, and treatment may be warranted in all patients undergoing transplant. From a diagnostic perspective, this study is also the first to document a drop in serum NGAL levels as early as one hour after transplant. Serum NGAL levels were markedly elevated and began to fall almost immediately after living donor renal transplantation. This decrease fits with the near instant urine output and rapidly falling creatinine clinically seen in living donor renal transplantation. Furthermore, high initial levels and rapid changes limit the utility of using any single NGAL measurement for the purpose of clinical prediction. Instead, an overall trajectory or relative change should be considered when using serum NGAL in patients with existing renal disease. Additionally, the percentage change in serum NGAL at one hour correlated well with the percentage change in creatinine 2 days after transplantation as well as the recipients' GFR at 90 days. These findings are consistent with those in previous studies and reinforce NGAL's utility as a marker for injury and short-term graft function in renal transplantation [18–20]. 

Of greater clinical importance, elevated glucose level at the time of reperfusion was inversely proportional to the percentage change in serum NGAL and creatinine. In other words, increasing blood glucose led to greater ischemia reperfusion injury as evidenced by changes in these markers. Even though NGAL levels fell in most patients, these levels fells less rapidly and even increased when the allograft was exposed to higher glucose levels at the time of reperfusion. Similarly, serum creatinine fells less rapidly with increasing blood glucose at the time of reperfusion. This phenomenon may imply that recipients with peri-operative hyperglycemia are at greater risk for DGF. As recipient blood glucose rises, the extent of ischemia reperfusion injury, and therefore the risk of DGF, may also increase. Overall, this pattern of injury is likely due to greater oxidative stress and inflammatory response, which fits with our animal model in which rats with hyperglycemia at the time of injury suffered higher terminal creatinine and greater acute tubular necrosis [11].

This relationship between hyperglycemia and ischemia reperfusion injury may explain, in part, the mechanism by which recipient diabetes leads to DGF. Although glucose levels increased both in diabetic and non-diabetic recipients during transplantation, diabetic recipients had much higher levels. Diabetes and blood glucose level at the time of reperfusion were clearly predictors of the percentage change in NGAL according to our univariate analysis. However, when we included both in our multivariate model, diabetes was no longer a statistically significant predictor. This finding supports the theory that hyperglycemia is the mechanism by which diabetes leads to DGF in kidney transplant recipients. Fortunately, this effect can be attenuated with greater attention to peri-operative glucose levels. Although targeting glucose levels <100 mg/dL can be difficult and potentially dangerous, our analysis demonstrates a potential benefit to any reduction in glucose level. Therefore, we believe that glucose levels should be checked in patients at risk for hyperglycemia and treated according to standardized protocols. 

Despite the strength of being a prospective study, our analysis has some limitations. Use of the living donor population allowed us to eliminate cold ischemia time and minimize variation in donor characteristics but prevented us from analyzing clinical outcomes such as DGF. Furthermore, the size of our population made it difficult to strictly control for every potential confounding factor, such as exact blood pressure throughout the case. The use of NGAL as a surrogate for ischemia reperfusion injury also raises a number of questions. While numerous studies link NGAL to graft function in renal transplantation, these studies vary in the type of NGAL tested (serum versus urine), the time points collected, and the method of comparison (absolute versus relative change in levels). We think that our use of the relative or percentage change in NGAL levels is critical in serum samples given the fact that patients with end stage renal disease have markedly elevated and variable levels at baseline. However, one clear metric for how to use NGAL and interpret it clinically has yet to be established. 

Overall, our study is a starting point to determine if peri-operative hyperglycemia has a clear effect on clinical outcomes in renal transplantation. We still need to determine if the observed effects of elevated glucose can be reversed with better glucose control. Similarly, if elevated glucose does increase ischemia reperfusion injury, it is not known how long the allograft is susceptible to injury or how long tight glucose control would be required to prevent injury. Most importantly, we need to determine if clinically significant endpoints like DGF and overall graft survival are clearly affected by intra-operative glucose levels. We have already begun to study the effect of glycemic control in deceased organ donors in prospective randomized trials and hope to extend this work to renal transplant recipients. 

## 5. Brief Summary

Diabetes has emerged as a risk factor for delayed graft function in renal transplantation. In order to elucidate the mechanism behind this association, we prospectively studied the effect of intra-operative glucose levels on neutrophil gelatinase-associated lipocalin (NGAL), a marker of acute injury, and creatinine in living donor renal transplant recipients. Both NGAL and creatinine were adversely affected by increasing glucose levels.

##  Conflict of Interests

The authors declare that they have no conflict of interests.

##  Disclosure

The results presented in this paper have not been published previously in whole or part, except in abstract form.

## Figures and Tables

**Table 1a:** (a) Univariate models of percentage change in NGAL.

Characteristic	*β* Coefficient	95% Confidence interval	*P*-value
Recipient age (per 10 years)	0.07	0.02 to 0.11	<0.01
Recipient gender (male)	0.12	−0.01 to 0.25	0.07
Recipient race (compared to white)			
Black	−0.13	−0.33 to 0.07	0.20
Hispanic	−0.14	−0.29 to 0.01	0.07
Asian/Pacific Islander	−0.08	−0.38 to 0.22	0.59
Blood glucose (per 10 unit increase)	0.02	0.01 to 0.03	<0.01
Blood glucose > 160 mg/dL	0.19	0.04 to 0.33	0.02
Recipient diabetes	0.18	0.07 to 0.30	<0.01
Panel reactive antibody	−0.12	−0.38 to 0.14	0.36
Recipient body mass index	0.01	−0.001 to 0.02	0.09
Warm ischemic time (>30 min)	0.14	−0.01 to 0.28	0.06
Intra-operative IVF (per liter)	0.03	−0.06 to 0.12	0.49
Thymoglobulin induction (compared to Simulect)	0.002	−0.15 to 0.15	0.97
Donor age (per 10 years)	0.05	−0.01 to 0.1	0.10
Donor gender (male)	−0.02	−0.15 to 0.12	0.79
Donor race (compared to caucasian)			
Black	−0.12	−0.33 to 0.08	0.24
Hispanic	−0.11	−0.27 to 0.05	0.18
Asian/Pacific Islander	−0.11	−0.37 to 0.14	0.38
Donor BMI	−0.005	−0.02 to 0.01	0.62

**Table 1b:** (b) Multivariate model of percentage change in NGAL.

Characteristic	*β* Coefficient	95% Confidence interval	*P*-value
Blood glucose > 160 mg/dL	0.19	0.06 to 0.33	<0.01
Warm ischemic time (>30 min)	0.11	−0.04 to 0.25	0.13
Recipient age (per 10 year increase)	0.05	0 to 0.10	0.05

**Table 2a:** (a) Percentage change in creatinine on post-operative day two.

Characteristic	*β* Coefficient	95% Confidence interval	*P*-value
Recipient age (per 10 years)	0.05	−0.02 to 0.11	0.15
Recipient gender (male)	0.11	−0.05 to 0.27	0.18
Recipient race (compared to white)			
Black	0.02	−0.23 to 0.26	0.88
Hispanic	−0.21	−0.29 to 0.02	0.03
Asian/Pacific Islander	−0.14	−0.51 to 0.22	0.43
Blood glucose (per 10 unit increase)	0.02	0.001 to 0.03	0.04
Blood glucose > 160 mg/dL	0.20	0.02 to 0.39	0.03
Recipient diabetes	0.16	0.001 to 0.32	0.05
Panel reactive antibody	−0.26	−0.58 to 0.05	0.10
Recipient body mass index	0.02	0.01 to 0.04	<0.01
Warm ischemic time (>30 min)	0.32	0.16 to 0.48	<0.01
Intra-operative IVF (per liter)	0.09	−0.01 to 0.20	0.09
Thymoglobulin induction (compared to simulect)	−0.20	−0.37 to -0.02	0.03
Donor age (per 10 years)	0.09	0.02 to 0.16	0.02
Donor gender (male)	−0.02	−0.15 to 0.11	0.79
Donor race (compared to caucasian)			
Black	0.04	−0.21 to 0.30	0.74
Hispanic	−0.15	−0.35 to 0.05	0.13
Asian/Pacific Islander	−0.08	−0.40 to 0.24	0.61
Donor BMI	0.002	−0.02 to 0.03	0.84

**Table 2b:** (b) Multivariate model of percentage change in creatinine on post-operative day two.

Characteristic	*β* Coefficient	95% Confidence interval	*P*-value
Blood glucose > 160 mg/dL	0.16	0.02 to 0.30	0.03
Warm ischemic time (>30 min)	0.23	0.07 to 0.40	<0.01
Recipient age (per 10 years)	0.06	0.002 to 0.1	0.04
Recipient BMI	0.01	−0.001 to 0.03	0.07
